# IMB-XMA0038, a new inhibitor targeting aspartate-semialdehyde dehydrogenase of *Mycobacterium tuberculosis*

**DOI:** 10.1080/22221751.2021.2006578

**Published:** 2021-12-02

**Authors:** Xiao Wang, Ruifang Yang, Sihan Liu, Yan Guan, Chunling Xiao, Chuanyou Li, Jianzhou Meng, Yu Pang, Yishuang Liu

**Affiliations:** a National Laboratory for Screening New Microbial Drugs, Institute of Medicinal Biotechnology, Peking Union Medical College and Chinese Academy of Medical Sciences, Beijing, People’s Republic of China; b Department of Bacteriology and Immunology, Beijing Key Laboratory on Drug-Resistant Tuberculosis Research, Beijing Tuberculosis and Thoracic Tumor Research Institute/Beijing Chest Hospital, Capital Medical University, Beijing, People’s Republic of China

**Keywords:** *Mycobacteria tuberculosis*, drug-resistance, aspartate-semialdehyde dehydrogenase, high-throughput screening, inhibitor

## Abstract

The emergence of drug-resistant tuberculosis (TB) constitutes a major challenge to TB control programmes. There is an urgent need to develop effective anti-TB drugs with novel mechanisms of action. Aspartate-semialdehyde dehydrogenase (ASADH) is the second enzyme in the aspartate metabolic pathway. The absence of the pathway in humans and the absolute requirement of aspartate in bacteria make ASADH a highly attractive drug target. In this study, we used ASADH coupled with *Escherichia coli* type III aspartate kinase (LysC) to establish a high-throughput screening method to find new anti-TB inhibitors. IMB-XMA0038 was identified as an inhibitor of *Mt*ASADH with an IC_50_ value of 0.59 μg/mL through screening. The interaction between IMB-XMA0038 and *Mt*ASADH was confirmed by surface plasmon resonance (SPR) assay and molecular docking analysis. Furthermore, IMB-XMA0038 was found to inhibit various drug-resistant MTB strains potently with minimal inhibitory concentrations (MICs) of 0.25–0.5 μg/mL. The conditional mutant strain MTB::*asadh* cultured with different concentrations of inducer (10^−5^ or 10^−1^ μg/mL pristinamycin) resulted in a maximal 16 times difference in MICs. At the same time, IMB-XMA0038 showed low cytotoxicity *in vitro* and *vivo*. In mouse model, it encouragingly declined the MTB colony forming units (CFU) in lung by 1.67 log10 dosed at 25 mg/kg for 15 days. In conclusion, our data demonstrate that IMB-XMA0038 is a promising lead compound against drug-resistant tuberculosis.

## Introduction

Although drugs such as rifampin, isoniazid, ethambutol have been regarded as the terminators of tuberculosis (TB) which claimed 9.9 million in 2020 [1], the emergence and spread of drug-resistant (DR) *Mycobacteria tuberculosis* (MTB) still pose a major threat to TB control programmes worldwide [2]. One of the main mechanisms conferring drug resistance is the mutations of the target genes [3–5]. Because the differences in topological structures between the mutant and the wild proteins are generally not obvious, it is difficult to develop new agents based on these verified targets for DR-TB. Recently, bedaquiline, delamanid and pretomanid have been endorsed for the treatment of DR-TB in view of its promising efficacy in clinical trials [6–9], which brings us optimistic news and motivates us to search for new anti-TB agents with novel mechanisms of action.

The cell wall of MTB exhibits markedly different patterns from other bacteria, composing of peptidoglycan, arabinogalactan, mycomembrane and capsular layer [10]. The integrity of the cell wall structure is essential for its survival, growth, permeability, virulence and resistance to antibiotics [11]. The peptidoglycan of MTB which provides anchor sites for the other cell wall elements is highly cross-linked [12], while *meso*-diaminopimelic acid (*meso*-DAP) is the core component in the linkage [13]. In MTB, *meso*-DAP is synthesized through the aspartate metabolic pathway that is absent in humans, and this pathway involves the biosynthesis of lysine, threonine, isoleucine and methionine [14]. The aspartate pathway is essential to the survival of tubercle bacilli [15], thus the key enzymes of this pathway are potential targets for the development of anti-TB drugs.

Aspartate semialdehyde dehydrogenase (ASADH), the second enzyme in this pathway, has been proved to be essential for the growth, morphology and pathogenicity of MTB [16]. Therefore, we consider *Mt*ASADH to be a promising new anti-TB target. In this study, a novel high-throughput screening model, *Mt*ASADH coupled with *Escherichia coli* type III aspartate kinase (LysC), was established to search for anti-TB drugs. 5-nitro-N-(5-(tetrahydrofuran-2-yl)-1,3,4-oxadiazol-2-yl)furan-2-carboxamide (IMB-XMA0038) which could inhibit MTB strains with various drug-resistant profiles potently, was obtained through this model. Furthermore, pharmacodynamic studies revealed that IMB-XMA0038 was a promising anti-TB candidate drug.

## Materials and methods

### Bacterial strains and growth conditions

MTB strains, listed in Table S1, were cultured in Middlebrook 7H9 broth supplemented with 0.2% glycerol, 0.1% polysorbate 80 and 10% ADC or 7H10 agar solid media supplemented with 10% OADC (ADC plus 0.003% oleic acid) [17]. Escherichia coli (E. coli) DH5α (TransGen Biotech, Beijing, China) and BL21 Star™ (DE3) (Invitrogen, California, USA) were cultured in Luria–Bertani (LB) broth or LB agar medium. Hygromycin (Amresco, Pennsylvania, USA) was added necessarily at 100 µg/mL for the cultivation of MTB. Ampicillin or kanamycin (Amresco, Pennsylvania, USA) was added at 100 µg/mL for the cultivation of E. coli. Chemical Reagents used in assays were purchased from Sigma-Aldrich (Sigma-Aldrich, Missouri, USA) unless stated separately.

### Plasmid construction

*E. coli* genomic DNA was extracted using Easypure genomic DNA Extraction kit (TransGen Biotech, Beijing, China), and MTB H37Rv genomic DNA was extracted according to previously described protocols [18]. The primers were designed using Primer 5.0 software for the amplification of *lys*C and *asadh* gene based on the sequences published in the NCBI database (GenBank accession number: 948531 and 885118). The primer sequences for amplifying *lys*C gene (1350 bp) were 5′-TTTTGAATTCATGTCTGAAATTGTTGTCT-3′ (*Eco*RI, sense) and 5′-AAAAAAGCTTTTACTCAAACAAATTACTA-3′ (*Hin*dIII, anti-sense). The primer sequences for amplifying *asadh* gene (1038 bp) were 5′-TTTTGAATTCATGGGCCTGTCAATAGGGATC-3′ (*Eco*RI, sense) and 5′ AAAAAAGCTTTCACAAGTCGGCGGTCAGC-3′ (*Hin*dIII, anti-sense). The PCR products were digested with *Eco*RI and *Hin*dIII and cloned into the corresponding sites of pET28a(+) respectively. The ligation mixtures were transformed into *E. coli* DH5α competent cells respectively, and sub clones were sequenced to confirm no mutations in these fragments. The correct plasmids were designated as pET28a(+)-LysC and pET28a(+)-ASADH.

### Expression and purification of recombinant protein

Plasmids pET28a(+)-LysC and pET28a(+)-ASADH were transformed into *E. coli* BL21 Star™(DE3) respectively to obtain the recombinant strains. Before the expression and purification of the two proteins, the induction time, temperature and the IPTG concentrations were explored to reduce the formation of a large number of inclusion bodies and increase the amount of the proteins. Finally, the LysC protein expression was induced with 0.5 mM IPTG at 30 °C for 4 h, and the *Mt*ASADH protein expression was induced with 10 μM IPTG at 23 °C for 12 h. The recombinant proteins were purified through Ni^2+^ ion-affinity chromatography using a linear gradient 40–400 mM imidazole in washing buffer (50 mM Tris–HCl, 300 mM NaCl, pH 8.0). The eluted fractions were analyzed by SDS-PAGE followed by Coomassie Blue staining. The protein concentration was measured using Easy II Protein Quantitative Kit (BCA) (TransGen Biotech, Beijing, China), and the purified proteins were stored at -80 °C with 50% glycerol.

### Activity of the enzymes

The reaction system to detect the activity of LysC was as follows: 50 mM Tris–HCl (pH 8.0), 5 mM MgCl_2_, 1 mM dithiothreitol, 0.5 mM aspartic acid (ASP), 0.5 mM ATP, 0.01 μg LysC and total volume was 100 μL. ADP-Glo™ Kinase Assay kit (Promega, Wisconsin, USA) was used to supervise the reaction by measuring the releasing of ADP [19].

In order to ensure that the subsequent coupling reaction went smoothly, LysC in the reaction system was increased to 0.1 μg, and the concentrations of ASP and ATP to 5 mM ASP and 2 mM ATP. NADPH was added up to 0.5 mM, and 0.01 μg MtASADH were added to initiate the reaction to detect the activity of MtASADH by measuring the variation of NADPH ultraviolet absorption at 340 nm using PerkinElmer Enspire 2300 Multilabel Reader (PerkinElmer, Massachusetts, USA).

Standard curves were made to convert the intensity of photoabsorption to the concentration of ADP or NADPH. The enzyme activity unit was defined as 1 U of the enzyme content which could consumed 1 μM substrate (or generated 1 μM product) every second under the condition described above. The effects of substrates on enzymes were proceeded as previously did [20]. Using the initial velocity as the ordinate and the concentration of substrate ASP, ADP and NADPH as the abscissa respectively, K_m_ and V_max_ values were determined by non-linear regression of the Michaelis–Menten model using GraphPad Prim 5.0 (GraphPad Software Inc., San Diego, CA, US). k_cat_ is the catalytic constant of the enzyme and was calculated as V_max_/(total concentration of enzyme).

### *Mt*ASADH inhibitor screening

150,000 synthetic compounds were screened at a final concentration of 20 μg/mL in order to screen out MtASADH inhibitors. The assays were carried out in the 96-well plates containing the following ingredients: 50 mM Tris–HCl (pH 8.0), 5 mM MgCl_2_, 1 mM dithiothreitol, 5 mM ASP, 2 mM ATP, 1 mM NADPH, 3 U LysC and 0.3 U ASADH. The MtASADH heat-inactivated group was used as a positive control, and the negative control group only contained DMSO. Z’-factor was calculated to assess the quality of the screening model [21]. The inhibition rate of chemicals was calculated as follows:

$$\;\text{\%}inhibition=\left(1-\frac{Ap-As}{Ap-An}\right)\times 100\text{\%}$$

in which, *Ap* and *An* represented the average absorbance of positive and negative controls respectively, and *As* was the absorbance of sample. Compounds were perceived as hits when the 30% inhibition limit was achieved. The IC_50_ value of the compound with the best inhibitory activity against *Mt*ASADH was determined at 0, 0.156, 0.313, 0.625, 1.25, 2.5, 5 μg/mL.

### SPR assay

The SPR assay was performed using CM5 chip (Reichert, New York, USA) with dextran and a Reichert 2SPR system in a PBST running buffer (pH 7.4) with 5% DMSO. CM5 chip was coated with purified *Mt*ASADH in 10 mM sodium acetate buffer at pH 4.5. To determine the binding affinity of *Mt*ASADH with IMB-XMA0038, solutions of compound (5% DMSO) at different concentrations (31.25–500 μM) were injected into the *Mt*ASADH immobilized chambers. The results were analyzed using TraceDrawer software (Reichert, New York, USA).

### Molecular docking between IMB-XMA0038 and *Mt*ASADH

To determine how the structure of IMB-XMA0038 contributes to its inhibitory activity against *Mt*ASADH, a molecular docking analysis between IMB-XMA0038 and *Mt*ASADH was performed using MOE 2014 software (Chemical Computing Group, Montreal, Canada). The crystal structure of *Mt*ASADH solved at a 1.95 Å resolution was retrieved from the Protein Data Bank (PBD: 3TZ6). 3TZ6 is a complex of *Mt*ASADH and inhibitor SMCs (Cys). The inhibitor SMCs (Cys) covalently binds around the catalytic site cys130, so the binding site of the inhibitor is defined as molecular docking pocket. IMB-XMA0038 molecular structure was optimized and then docked with the active pocket. According to the docking score, the result with the highest score was retained. The binding free energy of IMB-XMA0038 with *Mt*ASADH was calculated by MM/GBVI method of MOE software.

### Determination of the MIC of IMB-XMA0038 against MTB strains and conditional mutant MTB::*asadh* strain

The antimicrobial properties of IMB-XMA0038 were evaluated using the minimum inhibitory concentration (MIC) [22]. The compound was prepared in DMSO at the concentration of 10 mg/mL, and two-fold diluted from 64 μg/mL to 0.125 μg/mL using 7H9 broth with or without bacteria. INH was used as positive control, and the 7H9 broth medium containing the same DMSO concentration as blank control.

To confirm whether IMB-XMA0038 could interact with MtASADH, the assay was conducted by testing the sensitivity of conditional mutant MTB (MTB::asadh) to the compound, which was cultured in 7H9 broth with different concentrations of pristinamycin (10^−5^, 10^−1^ μg/mL) [16]. When the conditional mutant MTB was cultured in 7H9 medium supplemented with 10^−5^ μg/mL pristinamycin, MtASADH was slightly induced to express, and when it was cultured in 7H9 medium supplemented with 10^−1^ μg/mL pristinamycin, MtASADH was largely induced to express.

### Cell cytotoxicity assay

HepG2 cells (ATCCHB-8065) and Vero green monkey kidney cells (ATCCC1008) were used to test the cytotoxicity of the compound as previous described [23]. Cells were seeded in 96-well culture plates at a density of 5×10^3^ cells per well. MTT assays were used to detect the survival of cells which were exposed to different concentrations (3.125–100 μg/mL) of IMB-XMA0038. Absorbance was detected at 490 nm using PerkinElmer Enspire 2300 Multiabel Reader and the IC_50_ values were calculated using Graphpad prism 5.0.

### Acute toxicity *in vivo*

Six-week Kunming male mice were purchased from Beijing Charles River Laboratory Animal Technology (Beijing, China). The mice were randomly divided into three groups, including Control group (0.5% CMC-Na), IMB-XMA0038 (L) (500 mg/kg in 0.5% CMC-Na), and IMB-XMA0038 (H) (1000 mg/kg in 0.5% CMC-Na), with six mice in each group. Mice were administered via gastric gavage and closely monitored over 24 h. The number of surviving mice was recorded. All animal experimental procedures were performed under the regulations of the Institutional Animal Care and Use Committee of the Institute of Medicinal Biotechnology.

### *In vivo* activity

We used the SPF male Balb/c mice, 6–8 weeks old, 18–20 g, to test the anti-TB activity of IMB-XMA0038 *in vivo*. Eighteen mice were infected with about 100 CFU of H37Rv via 099C A4224 Inhalation Exposure System (Glas-col, Indiana, USA). The negative control group was treated with 5% carboxymethylcellulose (CMC)-Na (6 mice each group). IMB-XMA0038 was dissolved in 5% CMC-Na and was administered via gastric gavage at a dosage of 25 mg/kg/day. INH also administered at 25 mg/kg/day as the positive control. After administered 15 times (five times per week), mice were weighed and euthanized, while lungs of each group were homogenized in PBST and plated onto 7H10 agar for calculating CFU.

### Statistical analysis

Two tailed t-test as well as a nonparametric test implemented in Graphpad Prism5.0 were used to compare differences in paired groups at 95% confidence level.

## Results

### Expression, purification and characterization of enzymes

**Figure 1.**
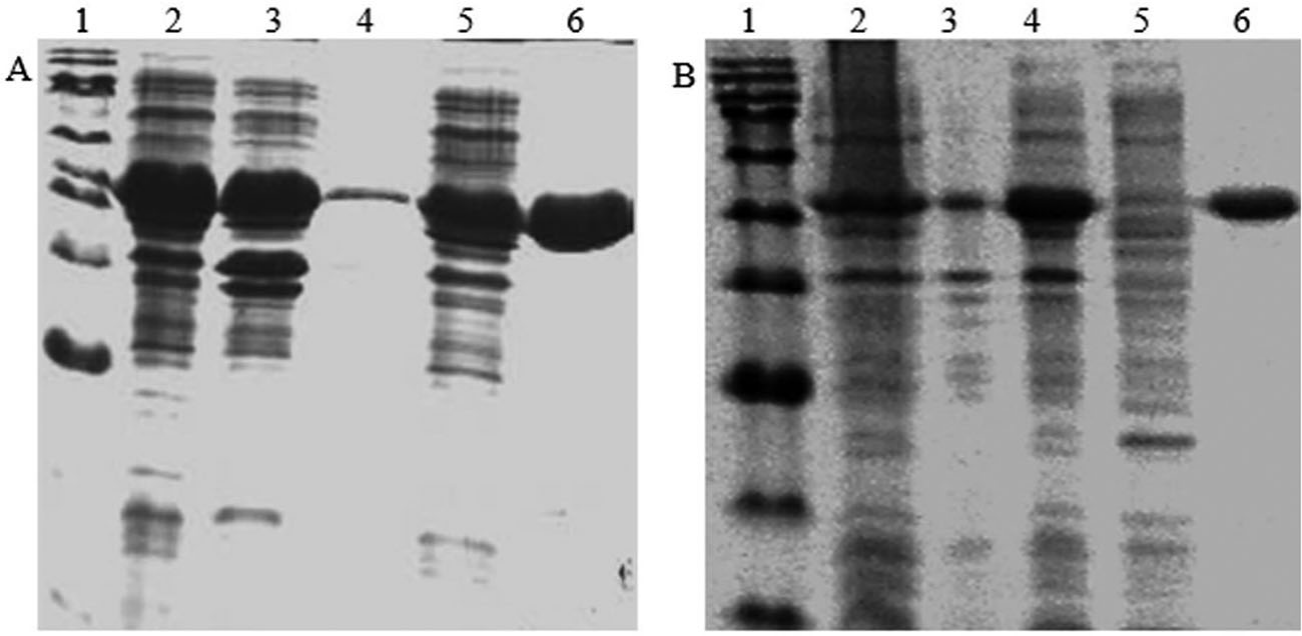
(a) The SDS-PAGE of the recombinant LysC. Lane 1: protein marker: 180, 135, 100, 75, 63, 48, 35, 25, 17, 11 kDa; lane 2: the whole protein of the recombinant cell; lane 3: cellular lysate supernatant; lane 4: precipitate protein; lane 5: the effluent liquid; lane 6: the purified LysC. (b) The SDS-PAGE of the recombinant ASADH. Lane 1: protein marker; lane 2: the whole protein of the recombinant cell; Lane 3: precipitate protein; lane 4: cellular lysate supernatant; lane 5: the effluent liquid; lane 6: the purified ASADH.

*LysC* and *asadh* were amplified by PCR from the genome of *E. coli* DH5α and MTB H37Rv, respectively (Figure S1). The LysC and ASADH recombinant proteins were purified using Ni^2+^ His Trap chelating columns. The purification was confirmed after SDS-PAGE and Coomassie blue staining (Figure 1). The molecular weight of the recombinant LysC protein was approximately 48.4 kDa, and that of ASADH was 38 kDa.

The activity of LysC (44.84 U/μg) was detected by measuring the production of ADP after incubating at 37 °C for 5 min. On the other hand, the activity of MtASADH (59.06 U/μg) was detected by measuring the absorbance corresponding to reduction of NADPH after 5 min of incubation at 37 °C (Figure S2). The influence of substrate concentrations on reaction rates was determined by changing the concentration of one substrate while fixing the other substrates. According to the initial reaction rates under different substrate concentrations, Michaelis–Menten equations were fitted through GraphPad Prism software (Figure S3). Since aspartyl phosphate is unstable to be quantified, we only studied the influence of NADPH on *Mt*ASADH. All these parameters were listed in Table S2, from which it could be concluded that the catalytic capacity of *Mt*ASADH was the limiting step in the LysC-ASADH coupling system.

### High throughput screening for *Mt*ASADH inhibitors

**Figure 2.**
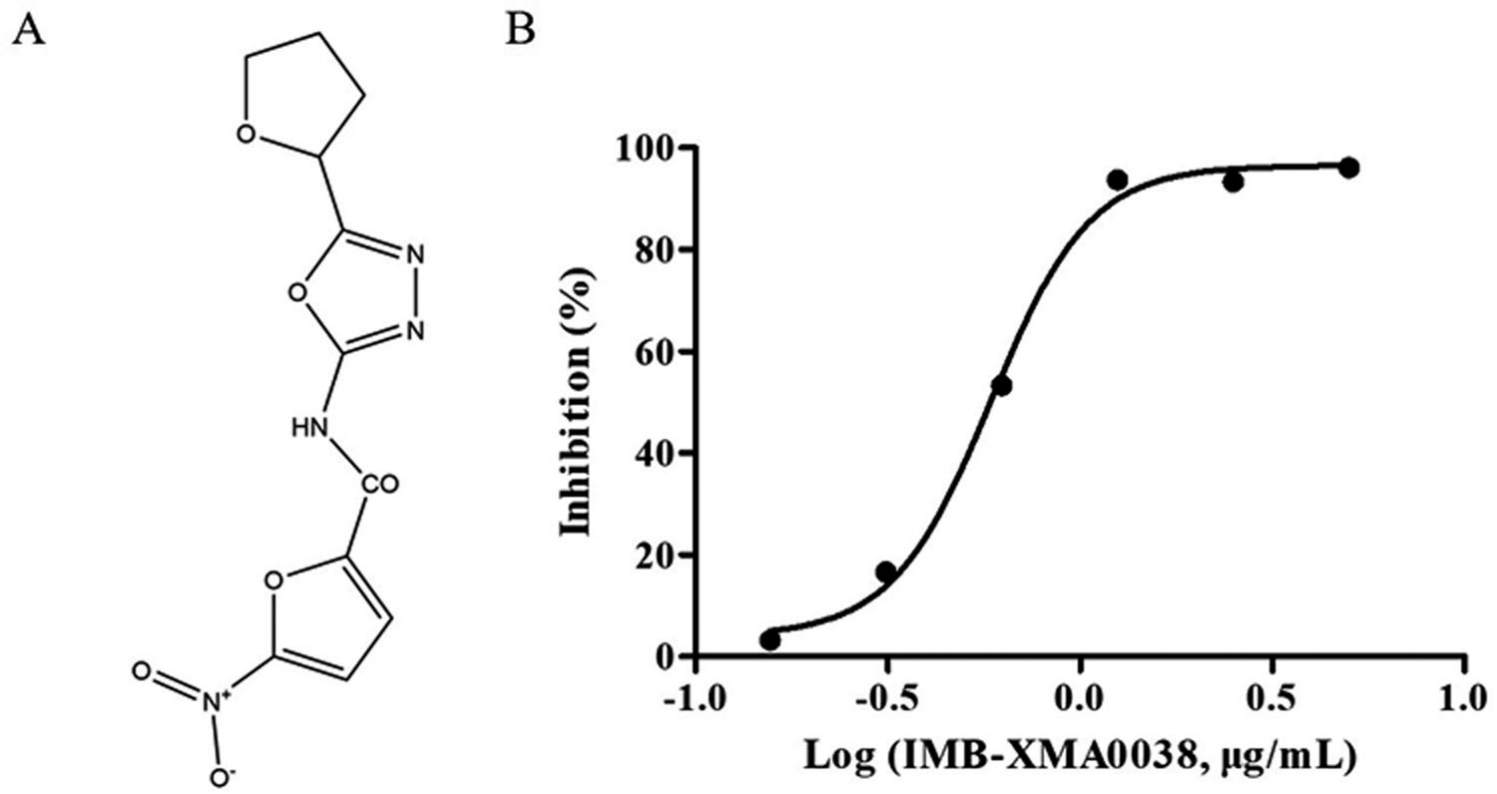
Identification of IMB-XMA0038 as an inhibitor of *Mt*ASADH. (a) The structure of IMB-XMA0038; (b) Inhibitory activity of IMB-XMA0038. The IC_50_ of IMB-XMA0038 was calculated from the inhibition rate of IMB-XMA0038 ranging from 0.156 μg/mL to 5 μg/mL with two folds dilution. Results were reported as mean ± SD (*n* = 3). IC_50_ was calculated using the log (inhibitor) vs. response-variable slope.

The high-throughput screening model was established based on the kinetic parameters of LysC and *Mt*ASADH. For obtaining the inhibitor of *Mt*ASADH, the dosage of LysC (3 U) was ten times of *Mt*ASADH. High concentrations of substrates (ASP and ATP, 5 × K_m_) were added to start the reaction rapidly, thereby producing large amounts of aspartyl phosphate. The model was evaluated with Z’-factor (0.77), which indicated that the reproducibility and sensitivity of the assay were reliable for high throughput. 143 compounds with an inhibition rate of ≥30% were identified. Among them, IMB-XMA0038 (Figure 2(a)) showed the best inhibitory activity, which could inhibit *Mt*ASADH in a dose-dependent manner with an IC_50_ of 0.59 μg/mL (Figure 2(b)). Its molecular properties were as follows: molecular weight (294), number of hydrogen-bonding acceptor (8) and hydrogen-bonding donor (1) and XlLogP (0.69), indicating no violations to Lipinski's “Rule of 5” [24].

### Confirming the interaction between IMB-XMA0038 and ASADH by SPR assay

**Figure 3.**
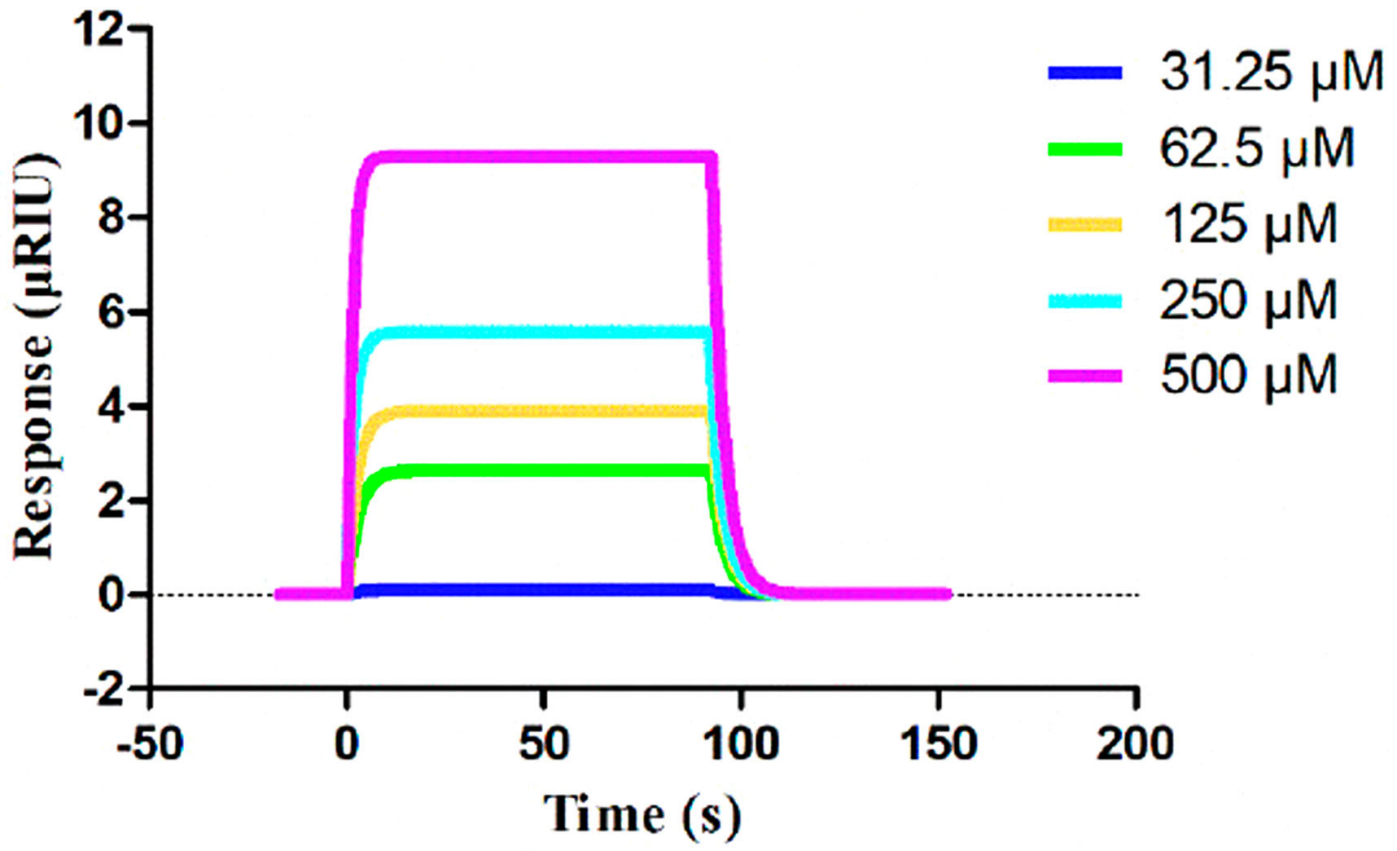
Demonstration of the interaction between *Mt*ASADH and IMB-XMA0038 by SPR. The figure was generated by SPR analysis software called TraceDrawer.

An SPR assay was used for examining the interaction between IMB-XMA0038 with *Mt*ASADH. CM5 chip was coated with *Mt*ASADH and then solutions of IMB-XMA0038 at different concentrations (31.25–500 μM) were injected into the *Mt*ASADH immobilized chambers. The result showed that IMB-XMA0038 was able to bind to *Mt*ASADH by a dose-dependent change (Figure 3). The affinity of the compound to *Mt*ASADH was identified by the K_D_ value, and the K_D_ is 3.65×10^−4^ M.

### Analysis of the molecular docking results

**Figure 4.**
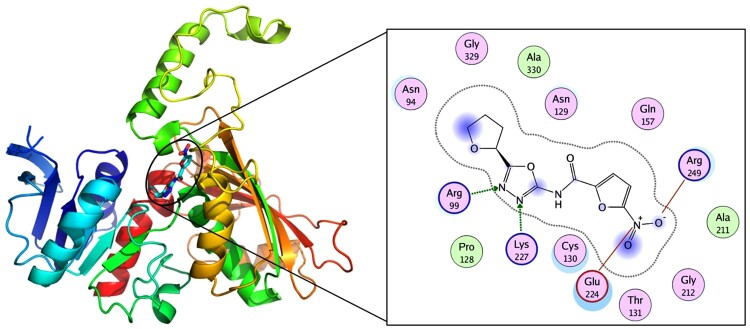
Molecular docking of *Mt*ASADH and IMB-XMA0038. The active pocket of *Mt*ASADH bound to IMB-XMA0038.

The ligand binds at the catalytic site of *Mt*ASADH around the residue Cys130. The nitrogen atoms on the [1,3,4] oxadiazole of the ligand form hydrogen bonds with R99 and K227. The nitro of the ligand forms salt bridges with the sidechains of E224 and R249. The ligand also forms polar interactions with N94, N129, C130, T131 and Q157 and hydrophobic interactions with P128 and A330 (Figure 4). The binding free energy between the ligand and *Mt*ASADH is -15.46 kcal/mol.

### Anti-MTB activity and MIC analysis over a conditional mutant MTB::*asadh* strain

We examined the anti-TB activity of IMB-XMA0038 against various MTB strains. As listed in Table 1, the MICs of MTB strains tested to IMB-XMA0038 ranged 0.25–0.5 μg/mL regardless of initial drug resistance profiles which was expected since the target was different from those of existing anti-TB drugs.

**Table 1.** MICs of IMB-XMA0038 against MTB and conditional mutant strain.

MTB strains	MIC of IMB-XMA0038 (μg/mL)	MIC of INH (μg/mL)
H37RV	0.5	0.125
FJ05349	0.25	0.125
FJ05060	0.5	0.125
FJ05195	0.5	>32
FJ05120	0.25	>32
FJ05189	0.25	>32
xz	0.5	>32
MTB::*asadh* (pristinamycin 10^−5^ μg/mL)	2	0.125
MTB::*asadh* (pristinamycin 10^−1^ μg/mL)	32	0.125

We further tested the susceptibility of the conditional mutant MTB::*asadh* to IMB-XMA0038. Our results showed that IMB-XMA0038 could inhibit mutant strains cultured in 7H9 broth containing 0.1 μg/mL pristinamycin at an MIC of 32 μg/mL, while it inhibited the strains cultured with 0.01 ng/mL inducer at 2 μg/mL, as low as one-sixteenth the MIC of those cultured with 0.1 μg/mL pristinamycin. Therefore, our experimental data indicated that IMB-XMA0038 could inhibit the growth of MTB via interacting with *Mt*ASADH.

Moreover, the cytotoxicity of IMB-XMA0038 was evaluated by its inhibiting capacity to HepG2 and Vero cells. The IC_50_ of cytotoxicity for HepG2 and Vero cells were >64 μg/mL, indicating that IMB-XMA0038 exhibited promising anti-TB activity without significant cytotoxicity. *In vivo* acute toxicity assay was performed using male Kunming mice. The surviving number of IMB-XMA0038 (L) group is 6/6 and that of IMB-XMA0038 (H) group is 5/6, indicating the safety of the compound in animals.

### Anti-tuberculosis activity of IMB-XMA0038 *in vivo*

**Figure 5.**
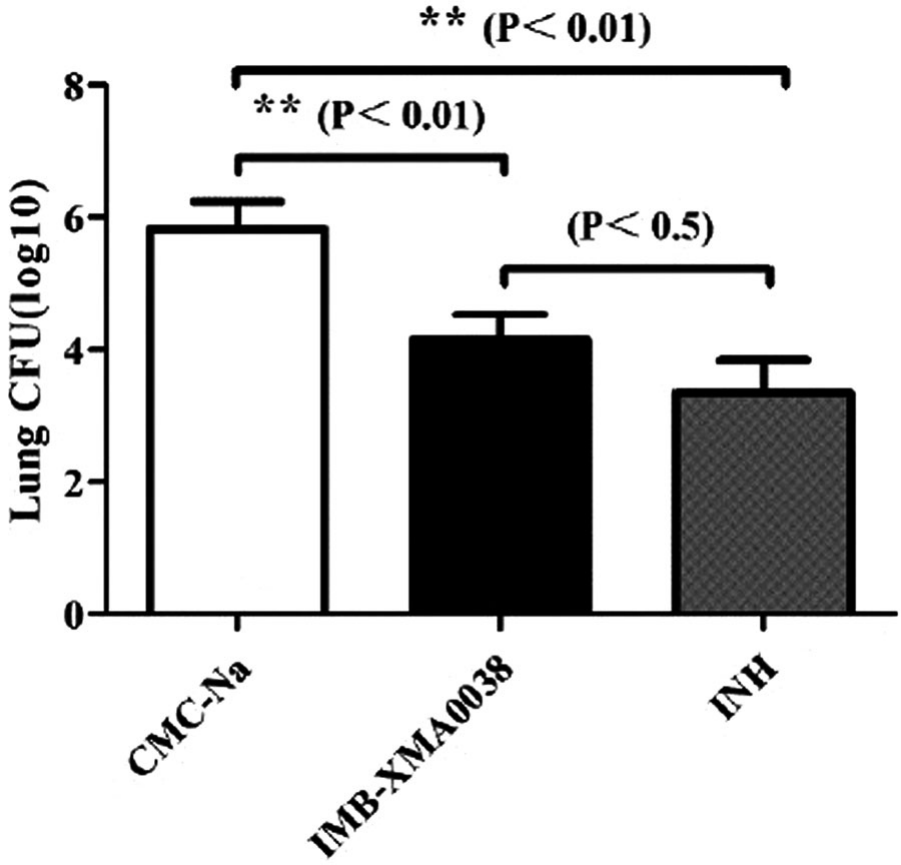
The activity of IMB-XMA0038 in BALB/C mice. INH and IMB-XMA0038 were both administered at 25 mg/kg respectively for 15 days, and the vehicle CMC-Na was used as negative control. **statistically significant difference from the negative control (*p*<.01) (*n* = 3).

The Balb/c mice acutely infected with MTB H37Rv for 10 days were administered IMB-XMA0038 at a dosage of 25 mg/kg/day, and INH were also administered at 25 mg/kg as the positive control. After 15 days of administration, the mice were weighed to count the MTB-CFU in the lungs for comparing the treatment effects. The body weight of all mice changed slightly, but there was no significant difference (*p*>.85) (data was not shown). However, the bacteria load of the mice treated with IMB-XMA0038 was greatly reduced by 1.67 log10 (*p*<.01) comparing with the negative control group (mice were treated with carboxymethylcellulose (CMC)-Na) (5.83 log10) (Figure 5). The bacteria load of the positive group was reduced by 2.48 log10. The results showed that the chemical IMB-XMA0038 could significantly inhibit the growth of MTB *in vivo*.

## Discussion

Aspartate metabolic pathway is an important way of antibacterial drug development [25,26], which is also crucial for the survival and pathogenicity of MTB [15,27]. This pathway is absent in humans, so the enzymes involved in this pathway are implicated as potential antibacterial targets. Previous studies showed that multiple genes of this pathway, such as dapA, dapB, and dapE, have been studied as targets for anti-TB drug development [28–30]. ASADH, occupying the first branch position of the biosynthetic pathway of the aspartate family of amino acids, also became an attractive target for antimicrobial agent development. Various phosphonamidites analogues of the substrate aspartyl phosphate have been synthesized and reported as inhibitors of *E. coli* ASADH [31].

Gao and colleagues established the first molecular assay to identify the ASADH inhibitors of *Streptococcus pneumoniae* (SP), *Vibrio cholerae* (VC) and *Candida albicans* (CA), by screening from a fragment library containing 378 compounds diluted as 94 cocktails [32]. They further eliminated the ineffective and toxic compounds, and finally found potent and selective ASADH inhibitors subsequently [33,34]. At the same time, molecular modelling and docking approach was used to identify the ASADH inhibitors of *Streptococcus pneumoniae* and *Vibrio cholerae* [35]. Kumar, R. *et al* used shape-based or ligand-based virtual screening methods to screen the ZINC and NCI databases for inhibitors of *Mt*ASADH [36,37], but they did not show whether the compounds could inhibit MTB *in vitro* and *in vivo*. However, the activity of the compounds obtained in silico should be verified at the biological level, as the biological process could not simply be computationally simulated. In this study, we established a high-throughput screening (HTS) model to search the inhibitors of *Mt*ASADH. 143 inhibitors were obtained from 150000 compounds, of which 107 showed high and moderate anti-tuberculosis activity *in vitro* (data was not shown). This is the first time that a biological HTS model is established to search for *Mt*ASADH inhibitors. The diversity of these active compounds will provide clue for the design and optimization of *Mt*ASADH inhibitors.

Additionally, we confirmed the interaction between IMB-XMA0038 and *Mt*ASADH by SPR assay. IMB-XMA0038 displayed a moderate affinity with the protein according to the K_D_ value (3.65×10^−4^ M), and the dissociation rate of the compound from *Mt*ASADH was fast according to the k_d_ value (3.01×10^−1^ s^−1^) (data not shown). Furthermore, we analyzed how IMB-XMA0038 inhibited the activity of *Mt*ASADH by molecular docking analysis. Cys130, forms a disulfide linkage with the inhibitor, is the active site catalytic residue in *Mt*ASADH [38]. Gln157 and His256 which located in the active site cleft are essential for catalysis [12]. Moreover, binding of the common active site inhibitor SMCS has been previously studied, suggesting that other residues (His256, Arg249, Glu224) in the active site also played essential role in ligand-binding [38]. Our molecular docking results showed that IMB-XMA0038 interacted with four of the above important amino acid residues (Arg249, Glu224, Cys130 and Gln157) of *Mt*ASADH, which comprised the binding site of its substrate (aspartyl phosphate or aspartate semialdehyde) [39,40], indicating the compound could interfere the binding of substrate to the enzyme. We speculated that IMB-XMA0038 bound to the active centre of *Mt*ASADH, competitively inhibited its catalytic activity, and then exerted antibacterial activity. In consistent to our hypothesis, the MIC value dramatically decreased with the downward expression of *Mt*ASADH, which indicated that *Mt*ASADH is likely to act as target of IMB-XMA0038. The SPR and molecular docking results provide a direction for the subsequent modification of IMB-XMA0038 to enhance the affinity and stability of binding to *Mt*ASADH, and improve its anti-bacteria activity.

IMB-XMA0038 showed strong anti-TB activity relative to INH *in vitro*, especially for drug-resistant MTB strains. We speculated that compound IMB-XMA0038 may inhibit the growth of MTB via a mechanism distinct from that of existing anti-TB drugs. Based on the above experimental results, we believe that the inhibitory activity of IMB-XMA0038 against *Mt*ASADH generated its anti-TB activity without cross-resistance of existing anti-TB drugs. Meanwhile, IMB-XMA0038 is less toxic at cellular and animal levels. Therefore, these results also show that IMB-XMA0038 merits chemical optimization for drug development. Moreover, *in vivo* experiments confirmed that IMB-XMA0038 had promising anti-TB efficacy. Further studies are urgently required to confirm the effect of IMB-XMA0038 on the integrity of the MTB cell wall, and assess its synergic activity in combination with other anti-TB agents.

In conclusions, we firstly obtained an excellent lead compound IMB-XMA0038, which could inhibit MTB targeting *Mt*ASADH with low toxicity, and further studies should be performed to improve its anti-TB activities and illuminate the mechanisms of inhibiting bacteria.

## Supplementary Material

supplementary_data.docx
