# Supplementary material for: IMB-XMA0038, a new inhibitor targeting aspartate-semialdehyde dehydrogenase of Mycobacterium tuberculosis
Source: Emerg Microbes Infect. 2021 Dec 2;10(1):2291–9. doi: 10.1080/22221751.2021.2006578 (PMC8648042; doi:10.1080/22221751.2021.2006578)
Supplement: supplementary_data.docx [file TEMI_A_2006578_SM1380.docx]

**Supplemental Tables and Figures**

**Table S1**

Table S1. The drug-sensitive background of MTB strains in this study

| MTB strains | Drug sensitive background* |
| --- | --- |

| H37Rv(ATCC27294) | INH(S), RMP(S), EMB(S), STR(S), CPM(S), KAN(S), OFX(S) |
| --- | --- |

| FJ05349 | INH(S), RMP(S), EMB(S), STR(S), CPM(S), KAN(S), OFX(S) |
| --- | --- |
| FJ05060 | INH(S), RMP(S), EMB(S), STR(S), CPM(S), KAN(S), OFX(S) |
| FJ05195 | INH(R), RMP(R), EMB(R), STR(R), CPM(S), KAN(R), OFX(R) |
| FJ05120 | INH(R), RMP(R), EMB(S), STR(S), CPM(S), KAN(S), OFX(S) |
| FJ05189 | INH(R), RMP(R), EMB(S), STR(S), CPM(S), KAN(S), OFX(S) |
| XZ | INH(R), RMP(R), EMB(S), STR(R), CPM(R), KAN(S), OFX(R) |

* INH, isoniazid; RMP, rifampicin; EMB, ethambutol; STR, streptomycin; CPM, cefpiramide; KAN, kanamycin; OFX, ofloxacin.

**Figure S1**


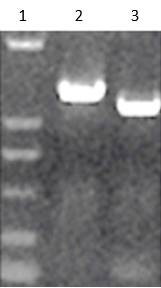


Figure S1 Agarose gel electrophoresis to confirm the PCR product of *lysC* and *asadh*. Lane 1: DL2000 marker: 2000 , 1000 , 750 , 500, 250, 100 bp; lane 2: *lysC* PCR product; lane 3: *asadh* PCR product.

**Figure S2**


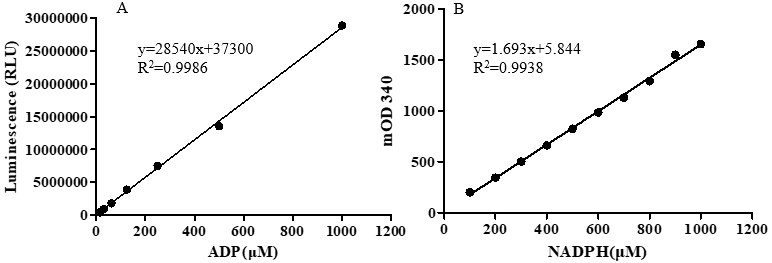


Figure S2 (A) The standard curve of fluorescence value to the concentration of substrate ADP. (B) The standard curve of absorbance value to the concentration of substrate NADPH.

**Figure S3**


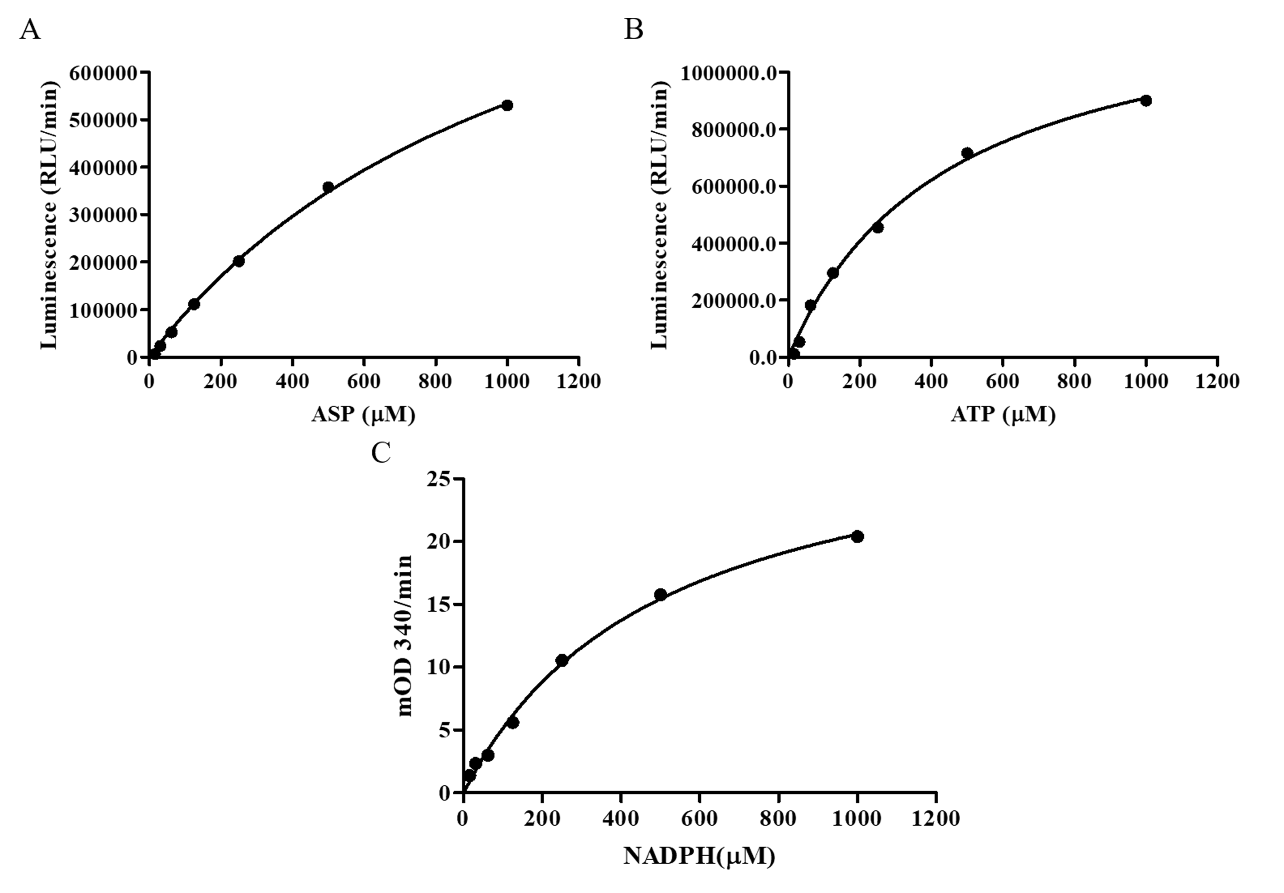


Figure S3 Calculation of the kinetics parameters of ASP (A) and ATP (B) to LysC, and NADPH to *Mt*ASADH (C) via Michaelis-Menten model.

**Table S2**

Table S2 Kinetic parameters for LysC and ASADH

| Enzyme substrate) | K_m_(μM) | V_max_^a^ (μM·min^-1^) | k_cat_^b^(min^-1^) | k_cat_/K_m_^c^(M^-1^min^-1^) |
| --- | --- | --- | --- | --- |
| LysC(ASP) | 1142±70.64 | 38.78±2.19 | 187.70 | 1.64×10^5^ |
| LysC(ATP) | 445.6±30.62 | 44.77±2.08 | 216.69 | 4.86×10^5^ |
| *Mt*ASADH (NADPH) | 497.8±31.55 | 14.76±1.65 | 54.61 | 1.10×10^5^ |

a: V_max_ was calculated by transforming the absorption rate to the substrate consuming rate via the standard curve of Luminescence or NADPH.

b: k_cat_ was calculated via $\bar{V}max/\left[ E \right]$.

c k_cat_/K_m_ was calculated via k_cat_ divided by $\bar{K}m$.
